# Apoptotic pathway protein expression variance in metal oxide and quantum dot treated HeLa cells

**DOI:** 10.17912/micropub.biology.000801

**Published:** 2023-07-18

**Authors:** Seth Harris, Kyoungtae Kim

**Affiliations:** 1 Biology, Missouri State University, Springfield, Missouri, United States

## Abstract

Using the HeLa cell line as a cancerous model, apoptotic protein expression was assessed upon various nanoparticle treatments. Utilizing a known chemotherapeutic agent, cisplatin, as a positive control for induction of apoptosis, several metal oxides (ZnO and CuO) and quantum dots (CdSe/ZnS and InP/ZnS) were investigated for their ability to express apoptotic markers. ZnO, CuO, green CdSe/ZnS, and green InP/ZnS were treated for 24 hours at their IC50 value. Western blot techniques were used to measure protein expression of phosphorylated p53 (ser15), PUMA, and p21 which are involved in signal transduction of apoptosis. CuO, ZnO, and CdSe/ZnS demonstrated considerable p53 activation at 24 hrs compared to the non-treated control. At the IC50 value, CdSe/ZnS quantum dots were the quickest at activating p53 by phosphorylation at the Serine 15 residue. Together, our results provide new insight into the apoptotic mechanism behind these treatments and lead to improved treatments against cancer.

**Figure 1. Nanoparticle-mediated cell toxicity and apoptosis induction:**
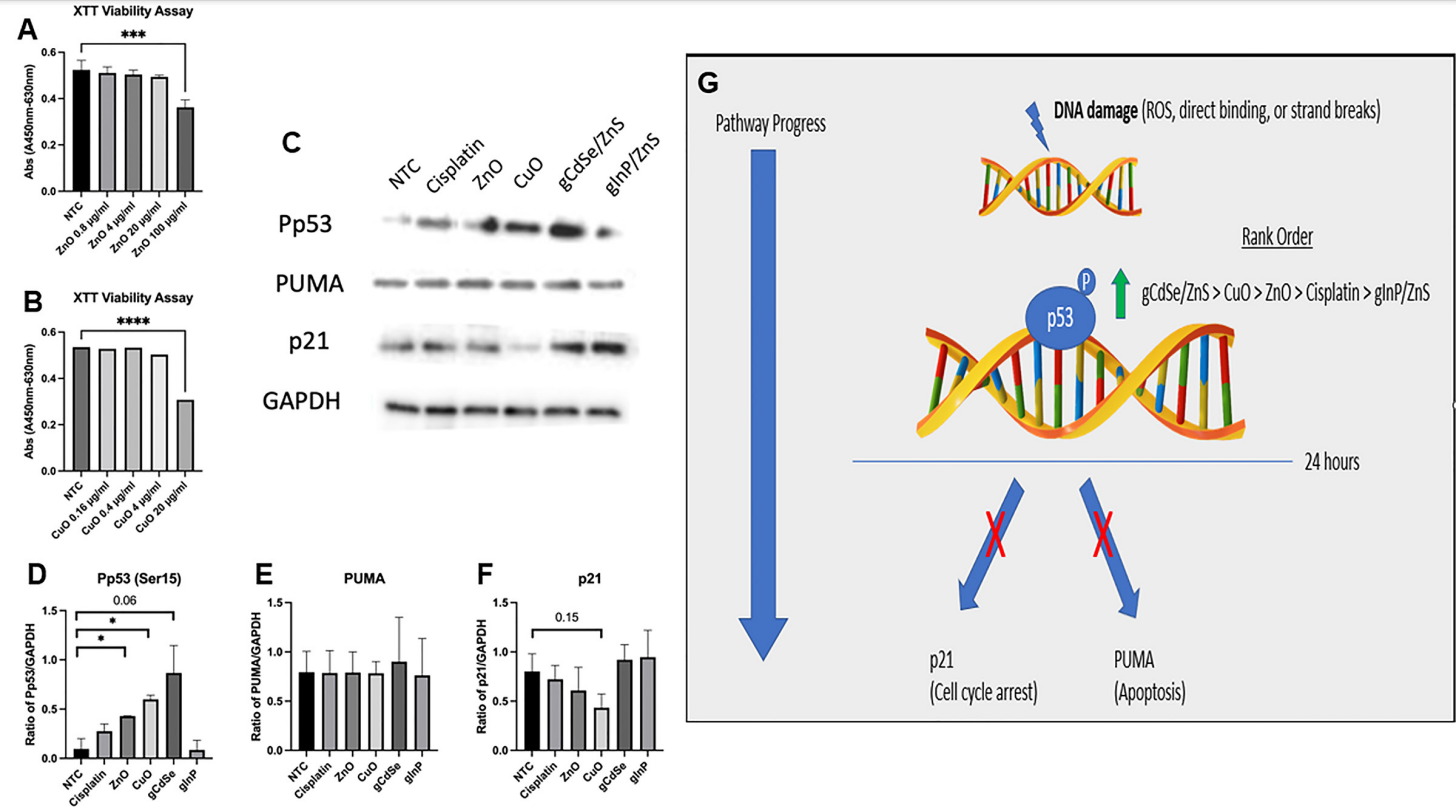
**XTT viability assay.** The effect of ZnO and CuO on the cell viability of HeLa cells. **A)** The average absorbance values for the ZnO-treated HeLa cell culture after 7 hours of incubation with activated XTT solution, and **B)** CuO-treated results with the same method. The absorbance values represent the delta wavelength range and lower values correlate to less cell viability. The treated cells were compared to the NTC using Prism8 software. A decrease in absorbance represents lower cell viability. The p values of *** represent <0.001 and **** <0.0001. **Western Blot Bands and protein/GAPDH ratios. C)** shows the representative protein bands for the triplicate western blot experiments. The phosphorylation of p53 at the serine 15 residue (Pp53) was examined as well as the downstream proteins PUMA and p21. GAPDH was used as a loading control. The treatment of HeLa cells was conducted for 24 hours at Ic50 values. Darker bands depict higher levels of protein expression. **D)** The ratio of Pp53/GAPDH. This ratio normalizes the data and reduces variation due to loading control errors. **E)** The ratio of PUMA/GAPDH. **F)** The ratio of p21/GAPDH. The intensity of the protein bands was measured using ImageJ software and averaged to get data for the figures above. Statistical analysis via Prism8 software compared the treated groups to the non-treated control (NTC). A * represents a p-value <0.05. **Representative** **Model. G)** A representative model of the results. The green arrow represents the protein elevation of phosphorylated p53 (Ser15). The red X’s indicate that the expression levels did not change for p21 and PUMA compared to the control. This model is applicable to the treatment of HeLa cells for 24 hours.

## Description

Metal oxide nanoparticles have been well studied regarding cytotoxicity. In particular, two metal oxide nanoparticles, CuO and ZnO, are found to be highly toxic (Wang et al., 2011) and have demonstrated anti-cancer effects in a variety of cancer cell lines (Vinardell & Mitjans, 2015) . A study comparing metal oxide toxicity in HepG2 cells demonstrated the cytotoxic abilities of CuO and ZnO and even found CuO to be more toxic than ZnO (Wang et al., 2011) . Another nanoparticle category of interest is quantum dots (QDs). QDs are semiconductors made stable with a shell that reduces leakage of metal (Zhang et al., 2022; Le et al., 2022) . Quantum dots are of interest in cancer-targeted therapy and bioimaging due to their ability to fluoresce when activated by light (Le et al., 2022) . The promising applications of QDs support the need to understand their effects. Generally, cadmium quantum dots (CdSe/ZnS) are seen to be more toxic than indium quantum dots (InP/ZnS), a possible safer alternative (Brunetti et al., 2013) .

In addition to cytotoxicity, the mechanism behind the cellular damage has also been well studied. The generally understood mechanism behind metal oxide toxicity is the elevation of ROS due to cellular stress which causes DNA damage (Benguigui et al., 2019) . A review detailing the literature around metallic nanoparticles and the role of ROS suggests that the surface chemistry of nanoparticles can potentiate the generation of ROS (Dayem et al., 2017). One specific study, testing CuO in HepG2 liver cells, found elevated oxidative stress to be the cause of DNA damage (Siddiqui et al., 2013) . Some metal oxides like ZnO have the potential to directly bind DNA. For example, ZnO can act as a cofactor affecting DNA damage repair and DNA replication (Alsagaby et al., 2020) . The severity of the damage will lead to either cell cycle arrest or cell death (apoptosis) (Benguigui et al., 2019) . With quantum dots, ROS is also elevated, which results in DNA damage (Davenport et al., 2021) . Research has found that the toxicity of CdSe/ZnS is due to Cd ^2+^ leakage as previously proposed (Manshian et al., 2017) . Other literature suggests that DNA damage is the primary mechanism for apoptosis induction in CdSe/ZnS quantum dots. Studies using plasmid DNA and treatment of CdSe/ZnS QDs in plant cells ( *Meticago sativa* ) demonstrated DNA damaging capabilities (Anas et al., 2008; Santos et al., 2013) . Heavy DNA damage triggers a cascade of proteins leading to apoptosis (Maclaine J. Nicola & Hupp R. Ted, 2009) . While the causes of cellular damage have been investigated, the subsequent pathway activation (specifically apoptosis) remains unknown.

The apoptosis pathway has key mediators that respond to DNA damage. Tumor suppressor protein p53 is an important mediator of the DNA damage signal and is phosphorylated most notably at the Serine 15 residue (Maclaine J. Nicola & Hupp R. Ted, 2009) . Upon activation of p53, downstream targets are engaged. Specifically, the PUMA (p53 upregulated modulator of apoptosis) protein which favors the continuation of apoptosis, or the p21 protein which favors cell cycle arrest (Maclaine J. Nicola & Hupp R. Ted, 2009) . PUMA works by antagonizing anti-apoptotic factors like Bcl-2 reducing the inhibition of pro-apoptotic proteins (Yu & Zhang, 2008) .Studies using radiation as a cell stressor have identified p21 arrests the cell cycle in the G1 phase (Abbas & Dutta, 2009) . If a treatment doesn’t induce enough of a stressor, pro-survival methods like cell cycle arrest should be favored.

While these nanoparticles have been studied regarding cytotoxicity and ROS production, a comparison between these nanoparticles on the level of proapoptotic protein expression is lacking. It would be beneficial to produce a model that tracks the differences in apoptotic pathway expression among treatments of different cancer-fighting nanoparticles including CuO, ZnO, CdSe/ZnS QD’s, and InP/ZnS QD’s. This allows for a better understanding of the molecular mechanisms and potential uses of these drugs in different types of cancer. To have a positive control, chemotherapeutic agent Cisplatin was used. This platinum-containing drug has been well documented to cause DNA lesions resulting in DNA damage responses (Murray & Mirzayans, 2020) .

Given the consensus that CuO and cadmium-based QDs are more toxic than ZnO and InP-based QDs, respectively, our working hypothesis is that CuO and CdSe/ZnS will elevate Pp53 (Ser15) and PUMA in favor of apoptosis at 24 hrs. On the other hand, ZnO and InP/ZnS, will favor an elevation of p21 for cell cycle arrest.

Therefore, the present study aims to uncover the subtle differences in apoptotic pathway expression among varied nanoparticle treatments. Understanding the differences in protein expression will help elucidate the apoptotic mechanisms for each treatment. This information could lead to the creation of a model that can comparatively determine the effectiveness of different anti-cancer agents at inducing apoptosis. This information will shed light on their usefulness as a personalized therapy for different types of cancer which may show varying degrees of sensitivity to metal oxide/quantum dot treatment.

*XTT cell viability results*

CdSe/ZnS and InP/ZnS toxicity assessment in HeLa cells determined that the IC50 values were 143 µg/mL for CdSe/ZnS and 97 µg/mL for InP/ZnS (Zhang et al., 2022) . Toxicity assessments of CuO and ZnO in HeLa cells were done elsewhere, however, we wanted to determine the IC50 for each. To determine the IC50 value for ZnO and CuO, a serial dilution was done. ZnO was diluted by factors of five starting with 100 µg/mL. Only at 100 µg/mL was there a significant decrease in cell viability indicating that ZnO becomes toxic somewhere around 20 µg/mL-100 µg/mL ( Figure 1A ). The serial dilution for CuO began at 20 µg/mL (lower stock concentration) and was diluted by a factor of 5 also. This assay revealed that CuO was able to reduce cell viability at 20 µg/mL but not at 4 µg/mL ( Figure 1B ). The concentration capable of reducing cell viability is somewhere between 4 µg/mL and 20 µg/mL. Using GraphPad Prism9, the calculated IC50 values were 46 µg/mL for ZnO and 13 µg/mL for CuO. In conclusion, all treatments have varying concentrations in which they induce toxicity. Knowing the IC50 values allows for the effects of CdSe/ZnS, InP/ZnS, CuO, and ZnO to be compared as they induce equal levels of toxicity.

*Western blot results*

The activation of p53 by phosphorylation can be induced by cellular stressors. DNA damage is known to induce phosphorylation of p53, specifically at the Ser15 residue (Maclaine and Hupp 2009) . DNA damage can occur through direct binding or oxidative stress. Therefore, the magnitude of p53 phosphorylation is directly proportional to the level of stress. Other stressors that can activate p53 include hypoxia and oncogene activation (Inoue et al., 2016) .

The expression of phosphorylated p53 (Pp53) at the Ser15 residue was highly elevated by ZnO, CuO, and CdSe/ZnS at 24 hrs ( Figure 1C ). Specifically, shown in figure 1D, we see that ZnO, CuO, and CdSe /ZnS are significantly expressing Pp53 compared to the non-treated control. Notably, InP/ZnS did not cause any elevation of Pp53 and was consistent with the control group ( Figure 1D ). While ZnO and CuO still elevated Pp53, CdSe/ZnS demonstrated the highest average expression of Pp53 ( Figure 1D ). After recognizing the elevation of Pp53, the downstream signals PUMA and p21 protein were investigated. In conjunction with the 24-hour treatment and use of IC50 values, none of the treatments had different expression of PUMA when compared to the control ( Figure 1E ). Similarly, p21 expression did not change among treated and non-treated groups ( Figure 1F ). Although, some variability in p21 expression was observed, on average the expression was not significantly different ( Figure 1F ). Overall, after 24 hours of treatment at IC50 values, only Pp53 (Ser15) demonstrated elevated protein expression. PUMA and p21 were not significantly elevated or decreased.

*A novel method for mechanistic comparison*

Using an expression ranking is a novel approach that allows for trends and connections to be made between treatments. The ranking orders the nanoparticles from greatest to least expression of Pp53 (Ser15). Few studies compare different nanoparticles to each other and instead focus on one nanoparticle with different cell lines. This method creates a clear picture of how safe these treatments are in comparison to each other. For example, the literature suggests that quantum dots are stable and safe (Derfus et al., 2004) , however, CdSe/ZnS demonstrated the highest p53 activation when compared to other toxic treatments like CuO, ZnO, and Cisplatin. Quantum dots have a variety of uses in biomedical applications including imaging and cancer-targeted therapy which is why their toxicity needs to be well understood (Gao et al., 2004) . Typical cytotoxic assays of these treatments are not protein-based and mainly look at cell viability and ROS. A benefit of this method is getting to compare different treatments side-by-side at the protein level. With this, the subtle differences between their toxic mechanisms can be found. With the large variety of nanomedicine available for research, it is surprising that there is little research comparing them. This study hopes to add new perspective and a different method for researching nanomaterials.

The limitations of this study include the detection limit for western blot. It is possible that PUMA and p21 had different elevations, but our western blot was not sensitive enough to detect the changes. There is also a chance for high error bars demonstrated by figure 1F . The level of variation can make the statistical difference difficult to interpret. In addition, early pathway activation (after 24 hours) was tested due to the fact that anytime longer than this caused too much viability decrease which resulted in less protein. Likely, 24 hours is not enough time for the signal cascade to reach PUMA or p21. Perhaps these treatments prefer a different route toward apoptosis. For example, the proteins Bax and Noxa are downstream targets of p53 that could be favored instead of PUMA (Sax & El-Deiry, 2003) .

In conclusion, a model ( Figure 1G ) has been created to demonstrate the results of these treatments on Pp53, PUMA, and p21 expression after 24 hours. In addition, a rank for the activation of p53 has been included to summarize the effects of the treatments on HeLa cells. This model gives new insight into the mechanism of toxicity for each treatment while directly comparing them. This approach helps to determine the usefulness of certain treatments in comparison to each other and understand the differences in their mechanisms.

## Methods

*Nanoparticles used for this study*

Cisplatin was purchased through Sigma-Aldrich and solubilized in 1X PBS at 1 mM concentration. Green CdSe/ZnS (Product #CZW-G-5) and green InP/ZnS (Product #INPW530-5) QDs were purchased from NN-Labs (Fayetteville, AR, USA) and suspended in water (1 mg/mL). Surface modifications for both QDs include carboxylic acid surface ligands. A ZnS shell contains the metal core (either CdSe or InP) improving stability and biocompatibility. Both QDs emit at 530nm ± 15 nm. The size of CdSe/ZnS ranges from 6.1-9.5 nm [9][9] and InP/ZnS ranges from 3.7-5.2 nm (Zhang et al., 2022) . ZnO and CuO nanoparticles were gifts from Jordan Valley Innovation Center (JVIC, Springfield, MO). Previous work using scanning transmission electron microscopy found the size of CuO to be 40 nm and ZnO to be 35-45 nm (Peters et al., 2023) .

*Antibodies for western blot*

All antibodies were purchased from Cell Signaling technology. The primary antibodies used were anti-GAPDH (Cat#, 2118S), anti-phosphor-p53 (Cat#, ser15, 82530S), anti-PUMA (Cat#, 98672S), and anti-p21 Waf1/Cip1 (Cat#, 2947S) as rabbit monoclonal antibodies. The secondary antibody used for all primary antibodies was an anti-rabbit IgG, HRP-linked antibody developed from a goat (Cat#, 7074S).

*Cell Culture*

The cervical cancer HeLa cell line was cultured in a 75 cm ^2^ flask with 10 mL Dulbecco’s Modified Eagle medium (DMEM) containing 10% fetal bovine serum (FBS) and 1% antibiotics (penicillin and streptomycin). The cell culture was kept in an incubator at 37°C with 5% CO _2_ . HeLa cells were grown until confluent and then used for seeding and treatment. To detach the cells, 3 mL of Trypsin w/ EDTA was added after the removal of the DMEM media. To neutralize the trypsin, 10 mL of fresh DMEM was added and pipetted up and down. The cells were then centrifuged at 400 rpm for 10 minutes until a pellet was achieved. The pellet was mixed with 10 mL of fresh DMEM, and the cell number was quantified using a hemocytometer.

*XTT Viability Assay*

Using the XTT cell viability kit from Biotium, the cell viability of ZnO and CuO-treated HeLa cells was assessed. On the first day, HeLa cells growing in a 25 cm ^2^ flask were detached and seeded into a 96-well plate. Ten thousand cells were seeded per well. Along with the cells, each well contained 100 µL DMEM (10% FBS and 1% antibiotics). The cells were allowed to attach overnight in a 37°C incubator with 5% CO _2_ . On day two the cells were treated with either ZnO or CuO and diluted with DMEM (total of 80 µL) to reach the desired concentration. The serial dilution for ZnO was for the following concentrations: 100 µg/mL, 20 µg/mL, 4 µg/mL, and 0.8 µg/mL. The serial dilution for CuO was for the following concentrations: 20 µg/mL, 4 µg/mL, 0.8 µg/mL, and 0.16 µg/mL. A non-treated control with 80 µL of DMEM was also included. Each concentration was completed in triplicate. The treated cells were incubated at 37°C with 5% CO _2_ for 24 hours. On the third day, the XTT reagent was created according to the manufacturer’s protocol (Biotium). The reagent was a combination of the tetrazolium salt XTT and an activating solution in a 200:1 ratio. 20 µL of the XTT reagent was added to each well and measurements were taken after 7 hours of incubation in a 37°C incubator with 5% CO _2_ . As tetrazolium salt is reduced by mitochondrial enzymes of active live cells, it turns into an orange product. This colorimetric assay was measured using a BioTek ELx880 plate reader and read at the delta absorbance range (450nm-630nm). All data were analyzed using GraphPad Prism9 software to find p values of <0.05 and calculate IC50 values.

*Western blot*

HeLa cells were seeded at 300,000 cells/well in a 6-well plate and allowed to attach overnight in DMEM. To prepare for treatment, the media was removed, and cells were washed twice with 1X PBS. The treatment was added based on their IC50 values (previously calculated) diluted with DMEM. After treatment, the plates were incubated for 24 hrs. Treatment was conducted at the following IC50 values: Cisplatin at 16 µM, ZnO at 46 µg/mL, CuO at 13 µg/mL, CdSe/ZnS at 143 µg/mL (Zhang et al., 2022) , and InP/ZnS at 97 µg/mL (Zhang et al., 2022) . The experiment was conducted in triplicate.

After incubation in the various treatments, cell lysate was extracted from the cells using a 1X protease inhibitor cocktail (Halt ^TM^ from Thermo Scientific) with additional 1X PMSF added in RIPA buffer (Cell Signaling Technology). The whole process is kept on ice to avoid damage to the proteins. The cells were then further lysed using sonification (Branson 2800) for 1 minute followed by 1 minute on ice. This cycle was repeated three times. Centrifugation of the lysed cells was performed at 14,000 rpm for 10 minutes at 4°C during which the supernatant was collected, and the pellet discarded. The collected protein was quantified via a Bradford assay in a 96-well plate. Bovine serum albumin (BSA) was used to create a standard curve and 2 µL of protein sample was used for quantification. Absorbances of the samples were read using an ELx808 plate reader at 595 nm and recorded on Gen5 software (BioTek). Fourteen µg of protein with SDS loading dye was loaded into an SDS-PAGE gel in 1X running buffer. After running the gel at 140v for 30 minutes, the gel was then transferred to a 0.45 µm pore size nitrocellulose membrane. The transfer occurred on ice in a transfer buffer running at 0.5A for 10 minutes on and off for 50 minutes. Ponceau-S staining was used to visualize the success of the transfer.

The membrane was then blocked using 5% milk in TBST (Tris Buffered Saline containing Tween 20). Primary antibodies were diluted in 5% non-fat dry milk in TBST and added to the membrane to be incubated overnight in a 4°C refrigerator. Secondary antibodies were incubated for one hour at room temperature. Radiance Q (Azure Biosystems), a chemiluminescent substrate, was added to detect bands. Specifically, the bands were imaged using an Azure Biosystems c300 gel imager. Statistical analysis was performed using ImageJ and GraphPad Prism9 software reporting a P value <0.05 as *. Image J was used to get a value for the intensity of the protein band images. Excel was used to calculate the ratio of experimental protein (Pp53, PUMA, p21) to control protein (GAPDH). First, the background was subtracted from the intensity of the protein bands. Then each experimental protein intensity was divided by the intensity of the control protein. This was to normalize the data and reduce variation due to sample loading errors.
